# Textural, rheological, and structural properties of turkey and chicken gelatins from mechanical deboning residues

**DOI:** 10.1002/fsn3.4143

**Published:** 2024-04-17

**Authors:** Abdullah Kurt, Omer Said Toker, Mehmet Akbulut, Hacer Coklar, Duygu Ozmen, Yilmaz Ozcan, Said Reza Hosseini, Furkan Turker Saricaoglu, Guntac Demir, Mustafa Samil Argun

**Affiliations:** ^1^ Department of Food Engineering, Aksehir Faculty of Engineering and Architecture Selcuk University Konya Turkey; ^2^ Department of Food Engineering, Faculty of Chemical and Metallurgical Engineering Yildiz Technical University Istanbul Turkey; ^3^ Department of Food Engineering, Faculty of Agriculture Selcuk University Konya Turkey; ^4^ Department of Food Engineering, Faculty of Engineering Kirklareli University Kirklareli Turkey; ^5^ Department of Food Engineering, Faculty of Engineering and Natural Sciences Bursa Technical University Bursa Turkey; ^6^ Erpiliç Integrated Poultry Production Marketing and Trade Bolu Turkey

**Keywords:** gelatin extraction, gelling, mechanically deboning residues, rheology, texture

## Abstract

Large amounts of collagen‐rich by‐products are generated in poultry processing. In particular, gelatin from the by‐products of turkey processing is limited. Gelatin extraction from turkey and chicken MDRs (mechanically deboning residue) was the purpose of this study. Both materials were modified at the highest swelling pH for chemical denaturation of collagen and hot water extraction of gelatin was performed at the optimum temperature–time, which was determined to be pH 1.0 and 80°C–6 h, respectively. In these conditions, yields of 9.90% turkey gelatin (TG) and 13.85% chicken gelatin (CG) were produced. They demonstrated similar viscosity, gel strength, and lightness values of 72–73 g, 2.5–2.7 mPas, and 31, respectively. These results are close to those of bovine gelatin (BG). TG with 239.78 g Bloom exhibited higher strength than CG (225.27 g) and BG (220.00 g). The melting and gelation temperatures of CG and BG were 21 and 30°C, respectively, while those of TG were 19 and 28°C. Imino acids (proline + hydroxyproline) of TG (22.82%) were higher than those of CG (20.73%). Fourier transform infrared spectroscopy (FTIR) analysis revealed secondary structure and functional groups of CG and TG similar to those of BG. CG displayed a higher thermal transition temperature than BG, while TG exhibited the highest temperature sensitivity, according to the differential scanning calorimetry (DSC) analysis. In conclusion, TG showed higher potential for effective utilization with higher bloom and imino acids. Overall, turkey and chicken MDRs are a promising and potential alternative source to produce gelatin with comparable properties to bovine gelatin for intended food applications as well as for pharmaceutical and cosmetic fields.

## Graphical abstract

This is a novel research paper on the production of gelatin from alternative sources, turkey and chicken MDRs (mechanically deboning residues), as by‐products of poultry processing. The findings of this study will be useful to the literature and the food science and technology.

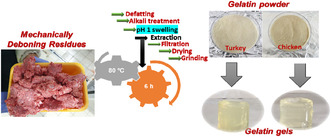

## 1 INTRODUCTION

Gelatin, as a water‐insoluble form of hydrolyzed animal‐derived collagen protein, is an important gelling agent that is thermally reversible, water‐soluble, and melts under conditions close to body temperature. In addition to the formation of edible films and improvement of textural properties by binding water, surface activity, foaming, and acting as a microencapsulation agent, gelatin is also crucial in the pharmaceutical, cosmetic, and health sectors. The amount of gelatin required is therefore constantly increasing. The worldwide requirement for gelatin increased by around 30%, from 350,000 tons in 2011 to 450,000 tons in 2018, and 620 kilotons was produced in 2019 (Abedinia et al., 2020). Research into appropriate alternative collagen raw materials is crucial to meet the ever‐increasing demand for gelatin.

Pigskin accounts for 40%–50% of the gelatin production volume. Bovine skin and bones have the same proportion, while other sources (poultry, fish, etc.) account for the remaining 1%–2%. While gelatin from pig waste is not preferred by some belief groups (Islam, Judaism, Hinduism), there is a risk of BSE (Bovine Spongiform Encephalopathy) prion carrier on bovine collagen. Therefore, there is increasing interest in producing gelatin from nonmammalian collagen to meet the growing demand and concerns of this consumer group. The lower gel strength, melting point, and stability of gelatin produced from fish waste by different extraction methods compared to mammalian gelatins and the presence of undesirable fish odor limit the use of fish gelatin (Rafieian et al., 2013). Poultry waste is considered to be a more important source of collagen. Poultry waste gelatin was found to be similar to mammalian gelatin in amino acid concentration, secondary protein structure, and molecular weight (Mhd Sarbon et al., 2013). Previous studies showed that poultry by‐products, such as chicken/turkey heads, bones, skin, and mechanically deboning residue (MDR), are important sources of collagen (Abedinia et al., 2020; Erge & Zorba, 2018). Mechanical separation is one of the main processing operations for recovering poultry meat and soft tissues from the bony waste fraction, excluding the final product, by applying pressure and named as mechanically deboning residue (MDR). It has been stated that the waste after this process contains about 20% protein and bone, skin, cartilage fragments, and 30%–40% collagen, which is the precursor of gelatin (Rafieian et al., 2013). The major steps taken in converting collagen into gelatin are conditioning with chemical denaturation and hot water extraction (Mokrejš et al., 2021). So, researchers aim to estimate the effects of these factors and optimize the gelatin production conditions.

Homogeneous components consisting of skin only or bone only were pretreated with increasing concentrations of acidic or alkaline solutions for increasing periods of time to break down collagen cross‐links as well as to eliminate non‐collagenous proteins and other contaminants (Du et al., 2013). These modification methods used to produce Type A and B gelatins are also applied to chicken MDRs (mechanically deboning residues), as described in the literature. Gelatin extraction was performed by Erge and Zorba (2018), who reported a gelatin yield of 15.34 g/100 g dry weight, from chicken MDR denatured at different sodium hydroxide (NaOH) concentrations (2–4.2%) at 80°C–250 min. In another study, chicken MDR was denatured with 6.73% hydrochloric acid (HCl) and water extraction was performed at 86.8°C for 1.95 h, resulting in a predicted yield value of 10.2% (Rafieian et al., 2015). However, when the raw material consists of heterogeneous mixtures like bone, skin, and cartilage as in MDR, different pretreatment studies are required. In addition, MDR is a by‐product of exposure to more severe process conditions (such as pressure). This indicates a high sensitivity to the higher concentrations of acid–base applications, thus swelling is one of the important factors for gelatin manufacture and which affects the gelatin properties, such as yield and gel strength (Kim et al., 2020; Park et al., 2013). The rate of swelling of duck skin in soaking solutions of different pH (1–12) was studied to determine the highest denaturation point of the non‐covalent bonds in collagen and the protein structure, and the highest swelling rate was observed at pH 1 by Kim et al. (2020).

Processing MDR to produce gelatin is not currently used industrially, as there are very limited studies addressing this issue for chicken and in particular turkey. There are studies in the literature on the use of skin and head raw materials for the production of turkey gelatin (Du et al., 2013; Ozcan et al., 2023), but MDR has not yet been studied for this purpose. Therefore, in this study, the pH condition with the highest swelling of the raw material was aimed to determine the hydrolysis of collagen prior to hot water extraction of gelatin, which is the first approach to produce gelatin from chicken and turkey MDRs. For the hot water extraction of gelatin, the optimum time and temperature were determined as the conditions under which the values of the dependent variables, such as yield, gel strength, viscosity, and clarity, were most similar to those of commercial bovine gelatin.

## 2 MATERIALS AND METHODS

### 2.1 Materials

Mechanically deboning residues (MDRs) of chicken and turkey were obtained at −18°C from a company (Erpiliç) located in Bolu, Turkey. The raw materials in frozen form were passed through a meat grinder to facilitate oil and other components' separation and also gelatin extraction. They were thawed at +4°C refrigerator conditions before extraction. Crude protein, fat, moisture, and ash analyses were performed within the scope of basic physicochemical analysis of the MDRs of turkey and chicken. The factor in protein analysis is 5.55. The results were obtained as percentage (%) of chicken and turkey MDRs on the wet matter with three replicates (Sila et al., 2017). The results are given in Table 1.

**TABLE 1:** Physicochemical parameters of raw materials (MDRs) and produced gelatins of chicken and turkey.

Parameters	Chicken MDR	Turkey MDR	Chicken gelatin	Turkey gelatin
Moisture (%)	61.05 ± 0.06^a^	60.66 ± 0.25^b^	6.78 ± 0.27^b^	7.21 ± 0.27^a^
Protein (%)	17.31 ± 0.06^b^	19.27 ± 0.18^a^	88.45 ± 0.01^a^	87.75 ± 0.01^a^
Fat (%)	11.56 ± 0.08^a^	7.74 ± 0.17^b^	2.47 ± 0.01^a^	1.83 ± 0.06^b^
Ash (%)	8.67 ± 0.05^b^	9.30 ± 0.22^a^	1.54 ± 0.62^a^	0.71 ± 0.09^b^

*Note*: (*n* = 3); Values are means ± standard deviation.

^a,b^Mechanically deboning residues (MDRs) and gelatins were compared within themselves.

### 2.2 Pretreatments applied to raw material

In the production of gelatin from turkey and chicken MDRs, similar pretreatments were applied to the raw materials. First, the raw materials passed through the meat grinder were mixed in diethyl ether at a ratio of 1:3 and kept for 4 days, and then diethyl ether was renewed again after 4 days to remove the fat. After oil separation, the drying process was carried out under room conditions. All subsequent gelatin production processes were carried out on dry raw material with oil and water removed. In order to remove the salt and alkali‐soluble parts of the proteins other than gelatin, a 1:4 (MDR:1% NaCl) mixture was stirred for 1 h at a pH of 10.5–11.00 and then the liquid part was removed by filtration through cheesecloth. Neutralization with distilled water was applied to the remaining solid phase (Erge & Zorba, 2018).

The maximum swelling point of the raw materials was determined by keeping turkey and chicken MDRs separately in pH 1–14 conditions prepared with pure water, 0.1 N HCl, and 0.1 NaOH solutions. It was kept in 1:5 MDR:liquid medium for 12 h at room temperature. After washing in running water, the swelling percentage analysis was calculated, according to the equation given below. The pH points at which the highest swelling was observed in 14 different pH conditions for both wastes were determined separately for turkey and chicken (Kim et al., 2020).

$$\text{Swelling}\;\left(\%\right)=\frac{\text{weight after swelling}-\text{weight before swelling}}{\text{weight before swelling}}\times 100$$

### 2.3 Gelatin extraction

**FIGURE 1:**
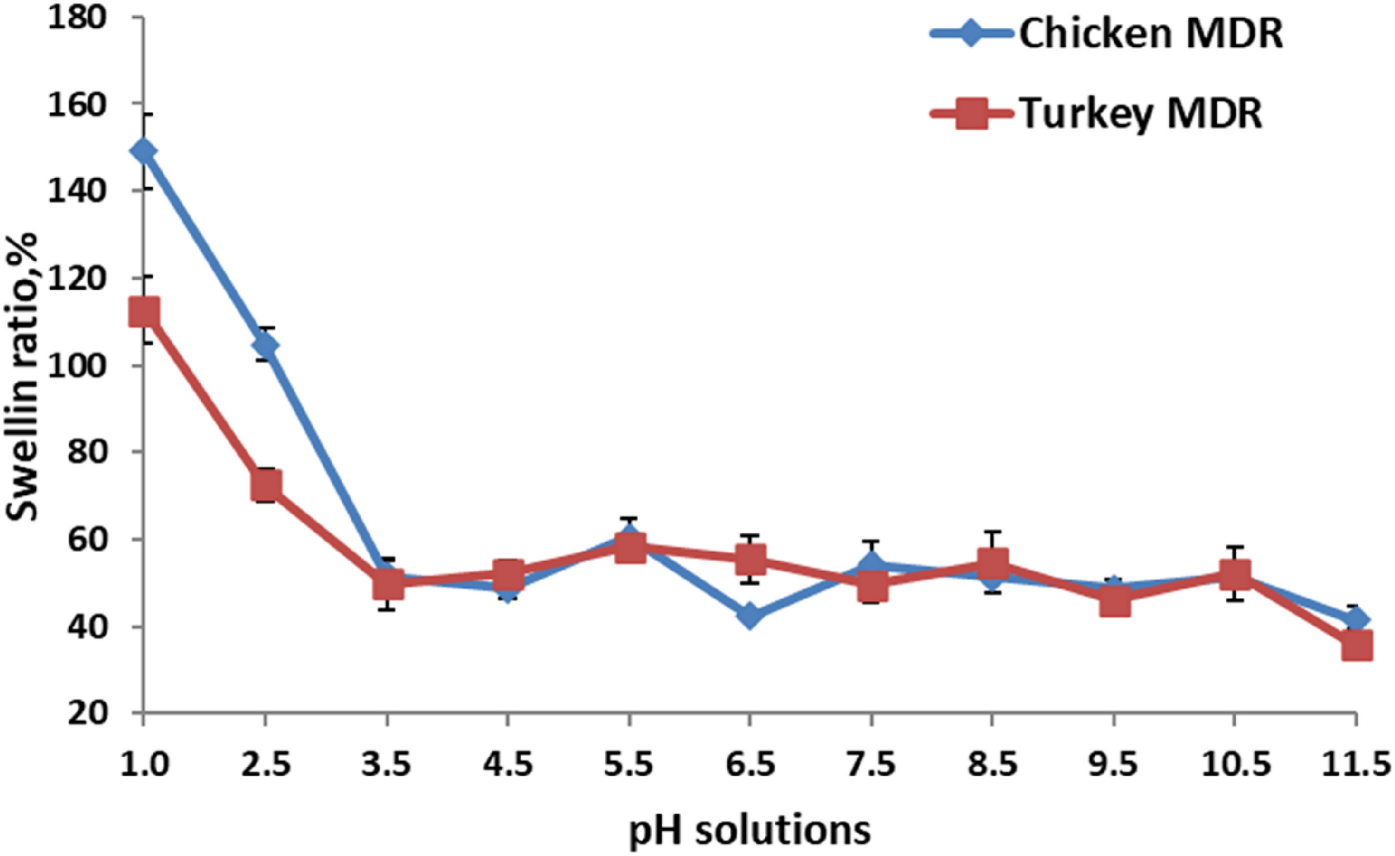
The swelling ratio of raw materials in various pH solutions.

The highest swelling value was observed in raw materials at pH 1 (Figure 1). Therefore, pH 1 condition was preferred for both raw materials in collagen modification. The solid phase was kept at pH 1 for 12 h and then neutralized by keeping it in pure water and 0.1 NaOH solution. For gelatin extraction, distilled water was added to the neutralized solid phase at a 10‐fold ratio and the extraction processes were carried out in a water bath mixer for chicken and turkey MDRs at 60, 70, and 80°C for 6 and 9 h. The solution containing dissolved gelatin was clarified by centrifugation at 10,000 × *g* for 30 min at 25°C and dried in a fan oven at 40–42°C and grounded. Yield, viscosity, gel strength, and color analysis were performed for the gelatin powders (Rafieian et al., 2013).

### 2.4 Analysis of gelatins

#### 2.4.1 Physicochemical analysis

The samples were analyzed for crude protein, fat, moisture, and ash using the standard method. The factor in protein analysis was 5.55 (Sila et al., 2017).

#### 2.4.2 Yield

The following equation was used for calculating the gelatin yield (Balti et al., 2011).

$$\text{Yield}\;\left(\%\right)=\frac{\text{weight of gelatin}}{\mathrm{wet}\;\text{weight of}\;\mathrm{MSM}}\times 100$$

#### 2.4.3 Gel strength (Bloom) and texture profile analysis

Gel strength (Bloom) was determined according to the standard method; the gelatin–water mixture at a concentration of 6.67% was kept at 10°C for 1 h. Gelatin was dissolved in a 60°C water bath for 30 min. The solutions within the jars were stored at 10°C for 16–18 h. The maximum force was determined as the Bloom value in grams using a 5 kg load cell and a 1.27 cm cylindrical probe (Erge & Zorba, 2018).

Texture profile analysis was performed on gels prepared by the same method using a cylindrical probe (P/36R) with a speed of 1 mm/s before, during, and after the test under conditions allowing 25% deformation. Before analysis, the gels were removed from the beaker. The data in the force–time graph obtained with two consecutive compressions were used to determine the hardness, adhesiveness, springiness, cohesiveness, gumminess, and chewiness parameters through the software of the device (Kurt et al., 2022).

#### 2.4.4 Color

The L* (whiteness or brightness) value of the gelatin solutions was determined using a Chroma Meter (CR‐400; Konica Minolta, Tokyo, Japan) (Ozcan et al., 2023).

#### 2.4.5 Rheological analyses

Viscosity–flow curve (steady) analysis was performed, where gelatin solutions (6.67% concentration) melted at 40°C for 10 min were analyzed at 0–100 s^−1^ shear rate for 3 min and the apparent viscosity was determined at 50 s^−1^ (Huang et al., 2018).

Temperature sweep tests were conducted at a constant stress and frequency of 1 Pa and 1 Hz, respectively, over a temperature range of cooling (50 → 10°C) and reheating (10 → 50°C) at a heating rate of 2 °C/min. Gelling (sol → gel transition) and melting (gel → sol transition) temperatures, which are the intersection points of elastic (*G'*) and viscous (*G*″) moduli during temperature decrease and increase, were determined. The time sweep test was carried out at +4°C to measure the rate and priority of gelation development with the profile of increase in elastic modulus (*G'*) (Kuan et al., 2016).

#### 2.4.6 Amino acid composition

The amino acid composition of produced gelatins (CG and TG) was analyzed according to the methods of Bilgin et al. (2019) using an Agilent 1260 Infinity HPLC system comprising of a binary pump, a degasser, and an autosampler coupled with an Agilent 6460 Series Triple Quadrupole Mass Spectrometer (Agilent Technologies, Santa Clara, CA, USA). Mass spectrometric detection was conducted using an Agilent 6460 tandem mass spectrometer (Agilent Technologies) equipped with electrospray ionization (ESI) in positive ionization modes.

Hydroxyproline was measured by the reported method (Alfaro et al., 2014). One hundred milligrams of gelatin was weighed into a 100 mL flask, 30 mL of 3.5 M sulfuric acid (H_2_SO4) was added, and the flask was sealed and hydrolyzed at 105°C for 16 h. From the filtered filtrate, 5 mL was taken out and further diluted to 100 mL. One milliliter of oxidant solution (chloramine T reagent) was added to the test tube, mixed in a vortex, and kept at room temperature for 20 min. Then 1 mL of the color reagent (4‐dimethylamino benzaldehyde) was added and mixed in a vortex. A spectrophotometer (Genesys 10S model, Thermo Scientific, Waltham, MA, USA) was employed to measure the absorbance at 558 nm. The concentration of hydroxyproline in the sample was determined as milligrams per milliliter (mg/mL) of hydroxyproline.

#### 2.4.7 Peltier system microscope images

The microstructure of gelatin solutions (6.67%) was studied using a PLM (polarized light microscope) (Carl Zeiss, NY, USA) fitted with a peltier system. The temperature was initially set at 40°C and then progressively reduced to 5°C at a rate of 2.5°C/min (Ozcan et al., 2023).

#### 2.4.8 ATR‐FTIR

Fourier transform infrared (FTIR) spectra of gelatin powders were determined by attenuated total reflectance (ATR)‐FTIR (Bruker Tensor 27 + HTS‐XT, Ettlingen, Germany). The wavelength range for the measurement was 4000–650 cm^−1^. Correction of the spectrum data (Data Tune‐Up) was performed with the software of the device (Zhang et al., 2020).

#### 2.4.9 Differential scanning calorimetry (DSC)

The thermal characteristics of gelatin powders were determined using DSC Q2000 (TA instruments). Five milligrams of the sample weighed into “aluminum pans” was scanned in the range of 10–90°C at a rate of 5°C/min (Giacomelli da Silva et al., 2022).

### 2.5 Statistical analysis

The data were evaluated using SPSS software (SPSS version 21.0, Chicago, IL, USA). All analyses were conducted in triplicate on duplicate samples. Student's paired *t*‐test analyses were performed in two groups. The mean and standard deviation of normally distributed samples were expressed, and the differences between samples were determined using one‐way analysis of variance (ANOVA), and Duncan's multiple range test was used to detect significant differences between means at a significance level of 0.05.

## 3 RESULTS AND DISCUSSIONS

### 3.1 Physicochemical properties of raw materials

Table 1 shows the results of the raw materials' physicochemical analysis. The protein content of the raw materials, which ranged from 17% to 20%, showed that they are important sources of gelatin, which was found to be higher for turkey MDR. The fat content of chicken MDR was higher than that of turkey. Drying was performed to remove both ether and water for extraction efficiency. More than half of the raw material components were water. Ash content was high and was found to be higher in turkey pulp due to the cartilage‐like components in the residue. A similar protein ratio but lower fat content was reported for chicken MDR by Erge and Zorba (2018). This suggests that pretreatment parameters for removing impurities could be varied according to the constituent of the raw materials, resulting in different gelatin yields.

### 3.2 Determination of the highest swelling pH

During the swelling process, the covalent bonds in the collagen structure are broken and the insoluble collagen is converted into a water‐soluble form and the yield increases accordingly (Park et al., 2013). The pH point of maximum collagen degradation was determined to be pH 1.0 for both MDRs, prior to water extraction (Figure 1). Chicken showed a higher swelling rate than turkey MDR. The highest swelling rate of duck skin was determined at pH 1.0 with a swelling rate of over 200% by Kim et al. (2020) who stated that Type A gelatins are produced under these conditions. For chicken and turkey MDRs, higher swelling was observed at low pH, probably due to their isoelectric points being in alkaline conditions (Park et al., 2013). Optimum swelling conditions for pork and skin were also reported at acidic conditions (Kim et al., 2020), but conditions for heterogeneous by‐products could be altered depending on fractional differences in raw materials. Therefore, prior to hot water extraction for higher yield, it is necessary to determine the maximum swelling condition of the raw material.

### 3.3 Gelatin extraction and optimization

The yield, viscosity, gel strength, and color results of six different turkey and chicken gelatins produced by gelatin extraction at various temperatures and times are shown in Table 2. The results were compared with those of commercial bovine gelatin as a control. The quality of gelatin was influenced by the temperature and time. The yields for turkey and chicken were found to be between 2.25%–12.10% and 5.03%–15.80%, respectively. Lower yields of gelatin were produced below 80°C. As expected, the increase in temperature and time provided an increase in yield (*p* < .05). High temperatures are more efficient at reducing the stability of interchain bonds in collagen and at breaking hydrogen and covalent bonds, causing more free α‐ and β‐chains to form and collagen to hydrolyze, thus increasing gelatin solubility and yield (Erge & Zorba, 2018; Sinthusamran et al., 2014). However, as in the chicken and turkey gelatins in this study, gel strength values decreased as the temperature and extraction time increased beyond the optimum conditions. Therefore, 6 h at 80°C was found to be the optimum extraction condition for both types of gelatins, where the yield was high (9.90% for turkey and 13.85% for chicken) and the gel strength and viscosity were similar to those of commercial gelatin (*p* > .05). The yields obtained here were lower than the values found (15.34%) by Erge and Zorba (2018) but higher than the values of (10.2%) found by Rafieian et al. (2015) for chicken MDR. In another study, mechanically deboned chicken meat residues subjected to acid and alkaline pretreatment resulted in a gelatin yield of 12 g/100 g (Rammaya et al., 2012). The variation in results may be due to differences in the effectiveness of the method as a function of temperature and extraction time, pretreatment conditions including swelling pH, and differences in raw material composition. The yield of chicken gelatin was found to be higher than that of turkey gelatin. Mechanically deboned chicken meat residue exposed to the acid and alkaline pretreatment resulted in a yield of 12 g/100 g. The color parameter *L*‐value ranged between 27 and 31. The *L‐*value of chicken and turkey gelatins was found to be lower than that of bovine gelatin (*p* < .05), which was associated with the difference in raw materials. Chicken and turkey gelatin extracts at higher temperature and times exhibited the highest brightness. At higher temperatures and times, increased hydrolysis of gelatin produces more free amino groups. These, in turn, may initiate a browning reaction along with carbonyl compounds (Ee et al., 2021). However, the *L‐*values of chicken and turkey gelatins were not varied in response to increases in time and temperature. As a result, in the remaining part of the paper, turkey and chicken gelatins produced at 80°C–6 h were characterized and compared with bovine gelatins.

**TABLE 2:** Extraction temperature and time conditions of gelatins.

	Yield (%)	Gel strength (g)	Viscosity (50 s^−1^) (mPas)	*L Lightness*		Yield (%)	Gel strength (g)	Viscosity (50 s^−1^) (mPas)	*L Lightness*
Bovine gelatin		77.91 ± 1.27^b^	2.25 ± 0.01^f^	38.14 ± 0.42^a^	Bovine gelatin		77.91 ± 1.27^c^	2.25 ± 0.01^e^	38.14 ± 0.42^a^
Turkey gelatin					Chicken gelatin				
60°C ‐6 h	2.25 ± 0.28^e^	118.24 ± 19.9^a^	12.37 ± 0.01^a^	29.60 ± 0.28^bcd^	60°C ‐6 h	5.03 ± 0.04^e^	100.36 ± 0.09^a^	5.37 ± 0.12^a^	29.66 ± 0.68^b^
60°C ‐9 h	3.05 ± 0.07^de^	96.86 ± 3.14^b^	8.95 ± 0.02^b^	30.27 ± 0.69^b^	60°C ‐9 h	7.63 ± 0.18^d^	91.48 ± 2.28^b^	3.19 ± 0.09^c^	29.87 ± 0.65^b^
70°C ‐6 h	4.09 ± 0.54^cd^	100.24 ± 4.82^b^	6.1 ± 0.01^c^	27.48 ± 0.13^d^	70°C ‐6 h	8.90 ± 0.14^cd^	79.78 ± 2.10^c^	4.08 ± 0.01^b^	29.57 ± 0.35^b^
70°C ‐9 h	4.51 ± 0.37^c^	85.39 ± 2.12^b^	3.69 ± 0.01^d^	27.70 ± 1.12^bc^	70°C ‐9 h	10.40 ± 0.57^c^	64.87 ± 1.34^d^	2.51 ± 0.06^d^	29.54 ± 0.27^b^
80°C ‐6 h	9.90 ± 0.14^b^	72.10 ± 0.53^b^	2.77 ± 0.00^e^	31.84 ± 0.89^cd^	80°C ‐6 h	13.85 ± 0.57^b^	73.78 ± 1.76^c^	2.50 ± 0.01^d^	31.18 ± 0.24^b^
80°C ‐9 h	12.10 ± 0.42^a^	33.67 ± 1.37^c^	2.12 ± 0.00^f^	30.27 ± 0.69^bc^	80°C ‐9 h	15.80 ± 0.28^a^	42.00 ± 0.61^e^	2.21 ± 0.04^e^	29.90 ± 0.44^b^

*Note*: (*n* = 3); Values are means ± standard deviation.

^a–e^Means within the same column with different letters are significantly different at *p* < .05.

### 3.4 Physicochemical properties of gelatins

Table 1 summarizes the physicochemical characteristics of turkey and chicken gelatins. The moisture content of the gelatin produced was found to be less than 10%. Low moisture content prevents gelatin powders from becoming sticky and extends their shelf life (Rahman & Jamalulail, 2012). The produced gelatins contain 87%–88% protein as the main component in dry matter. The protein content of fish, pig, and bovine skin gelatins was reported to be between 89.8% and 91.3% by Ninan et al. (2014). The presence of very high levels of protein and very low levels of ash, lipids, and other impurities are important factors for gelatin quality (Yahdiana et al., 2018). With the pretreatments applied, the amount of fat in the raw material decreased from 11.5% to 2.4% for chicken and from 7.7% to 1.8% for turkey, suggesting an efficient removal of fat. The gelatins contained less than 2% ash, indicating that the pretreatments significantly reduced the ash content in the raw material and resulted in higher quality gelatins (Uriarte‐Montoya et al., 2011). Above a certain level, the ash content may affect the gelling properties negatively. On the other hand, it can increase the water retention capacity of the gel system by forming cross‐links between the proteins and improving the gelatin properties (Mirzapour‐Kouhdasht et al., 2020). Rafieian et al. (2015) reported 4.41% ash for chicken gelatin produced from by‐products with acid pretreatment, while Mhd Sarbon et al. (2013) found 0.32% ash for chicken skin gelatin.

### 3.5 Gel strength (Bloom) and texture profile analysis (TPA)

Bloom strength and TPA results of gelatins are presented in Table 3. Bloom strength is an important criterion for determining gelatin quality. The gelatins produced in our study were in the high Bloom strength group (225–239 Bloom), which was higher than that of commercial bovine gelatin (220 Bloom). In addition to the gelatin extraction parameters, the proline and hydroxyproline content and the α/β chain ratio influence the gel strength (Ahmad et al., 2010; Mirzapour‐Kouhdasht et al., 2020). Therefore, the highest bloom strength values of the turkey gelatin (239 Bloom) were attributed to the higher content of proline and hydroxyproline. Considering bloom strengths of seafood gelatins as 100–120 g (Mirzapour‐Kouhdasht et al., 2020; Rafieian et al., 2015), and higher bloom of chicken and turkey than bovine, proved the importance of poultry MDR for gelatin production.

**TABLE 3:** Bloom strength and texture profile analysis (TPA) results of gelatins.

	Bloom strength (g)	Texture profile analysis (TPA)					
		Hardness (g)	Adhesiveness (N.s)	Springiness (mm)	Cohesiveness	Gumminess (g)	Chewiness (g.mm)
Chicken gelatin	225.27 ± 7.83^b^	588.1 ± 8.361^b^	−1.33 ± 0.27^a^	0.93 ± 0.00^a^	0.82 ± 0.00^b^	487.2 ± 11.5^b^	444.1 ± 12.3^b^
Turkey gelatin	239.78 ± 7.63^a^	592.37 ± 37.01^b^	−0.79 ± 0.25^a^	0.90 ± 0.081^a^	0.84 ± 0.05^b^	500.2 ± 8.9^b^	451.8 ± 0.7^ab^
Bovine gelatin	220.00 ± 2.12^c^	706.46 ± 21.13^a^	−1.44 ± 0.26^a^	0.80 ± 0.056^b^	0.99 ± 0.03^a^	699.8 ± 5.5^a^	561.1 ± 34.2^a^

*Note*: (*n* = 5); Values are means ± standard deviation.

^a–c^Means within the same column with different letters are significantly different at *p* < .05.

As a parameter of TPA, the hardness value of the produced gelatins was lower than that of the commercial gelatins. There was no statistical difference between the hardness values of the chicken and turkey gelatins (*p* > .05). There was no difference in the adhesiveness values between the gelatin samples (*p* > .05). Hardness and springiness are inversely correlated; as hardness increases, elasticity declines (Kreungngern & Chaikham, 2016) and no differences were found between the samples (*p* > .05), indicating similar deformability of the gelatin network between turkey–chicken gelatins and bovine gelatin (Hesarinejad et al., 2021). Cohesiveness is the parameter that is measured in relation to several factors, such as the strength of the internal structure of protein, the rate of deformation under mechanical stress, and the difficulty of breaking internal bonds (Mehrabani et al., 2022). There was no difference between the cohesiveness, gumminess, and chewiness values of chicken and turkey gelatins (*p* > .05), but their values were lower than those of bovine gelatin, suggesting that they are easier to chew.

### 3.6 Gelling and melting temperatures

**FIGURE 2:**
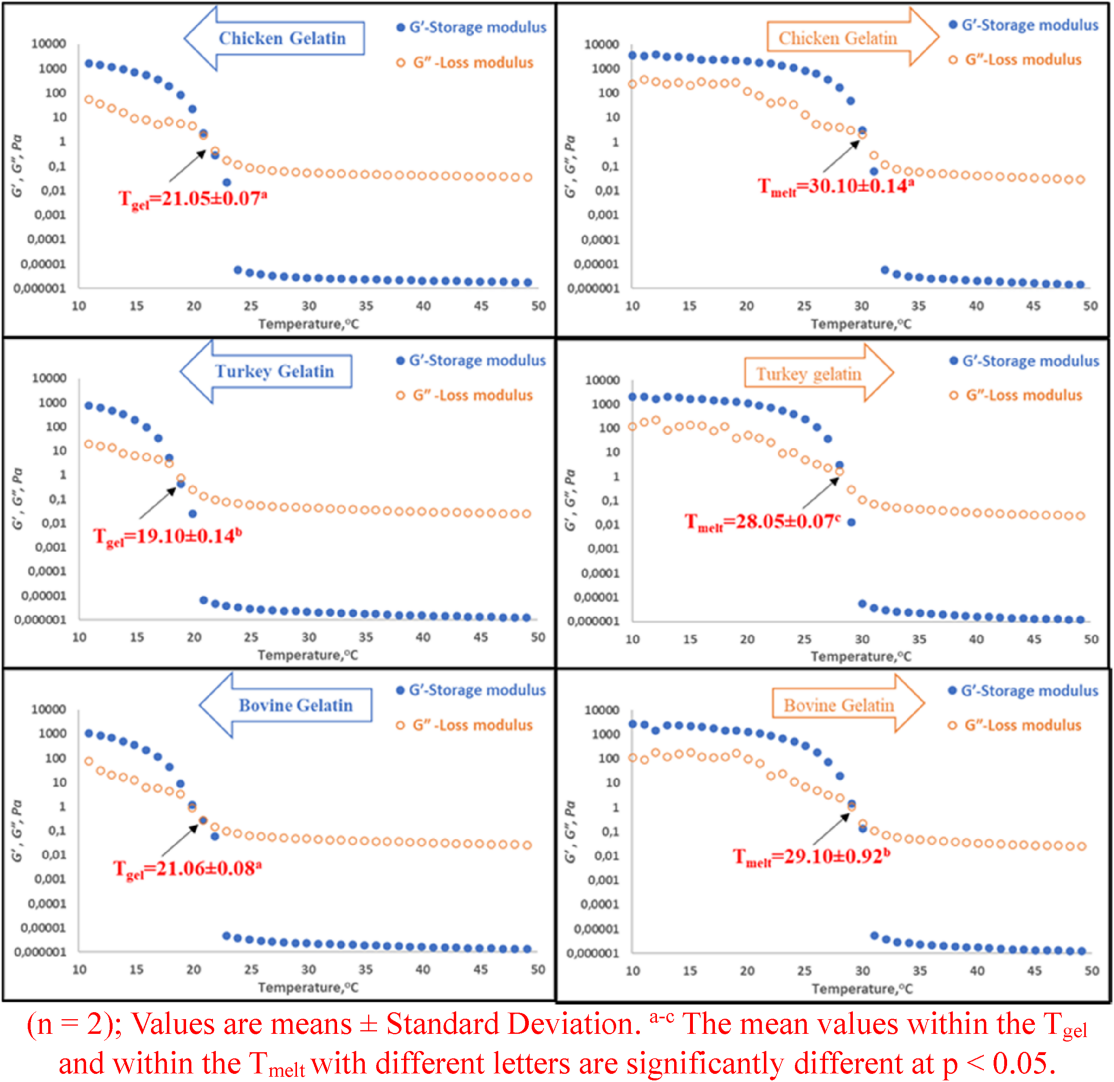
Gelling and melting temperatures of gelatins. (*n* = 2); Values are means ± standard deviation. ^a–c^The mean values within the *T*_gel_ and within the *T*_melt_ with different letters are significantly different at *p* < .05.

Figure 2 illustrates the gelling and melting temperatures measured during the cooling (*T*_gel_) and heating (*T*_melt_) periods at the crossover points of *G′* and *G′′*, respectively. *T*_gel_ values of turkey gelatin (19.10°C) are lower among the samples (*p* < .05), whereas those of chicken gelatin (21.05°C) are comparable to those of bovine gelatin (21.06°C) (*p* > .05). During cooling, the viscoelastic moduli increase abruptly due to the conformational transition random coil → helix, indicating that the temperature‐dependent network formation rate of chicken and bovine gelatins was similar and also earlier than that of turkey gelatin. When the gelation process is complete, a solid‐like property is found with an elastic modulus that is significantly higher than the viscous modulus at low temperatures. The microstructure is characterized by a network of well‐linked triple helices at this stage (Avallone et al., 2021), and similar *G′* and *G″* values at the end of cooling measurements demonstrate a similar network formation between chicken and turkey and bovine gelatins. High‐molecular‐weight components are associated with *G′* and an excess of low‐molecular‐weight fractions is associated with low *G″*. In the heating step, the process is reversed and the *G′* and *G″* values decrease as the temperature increases. Chicken gelatin melted at a higher temperature (30.10°C) than bovine gelatin (29.10°C), while turkey gelatin (28.05°C) melted at the lowest temperature. Chicken gelatin possesses favorable sensory properties because gelatin that melts at higher temperatures provides better mouthfeel (Sinthusamran et al., 2014). The amounts of proline and hydroxyproline, differences in molecular weight, and the relative content of α‐ and β‐chain components influence the thermal behavior of gelatins significantly (Abuibaid et al., 2020; Mohammadnezhad & Farmani, 2022). As the ratio of these amino acids increases, the melting and gelling temperatures also increase. However, this study revealed higher levels of both amino acids in turkey gelatin. The lower gelling and melting temperatures of turkey gelatin may be due to the higher formation of low‐molecular‐weight protein fraction during pretreatment for turkey gelatin (Erge & Zorba, 2018). Marine gelatins have a lower imino acid content than mammalian gelatins and are more prone to melting and gelling at relatively low temperatures, resulting in inferior commercial quality gelatins. The difference between the melting and gelling temperatures was approximately 9.05, 8.95, and 8.04 for chicken, turkey, and bovine gelatins, respectively. For certain food applications that require a large gap between the melting and gelling temperatures, chicken and turkey gelatins may be useful (Boran et al., 2010).

### 3.7 Time sweep tests

**FIGURE 3:**
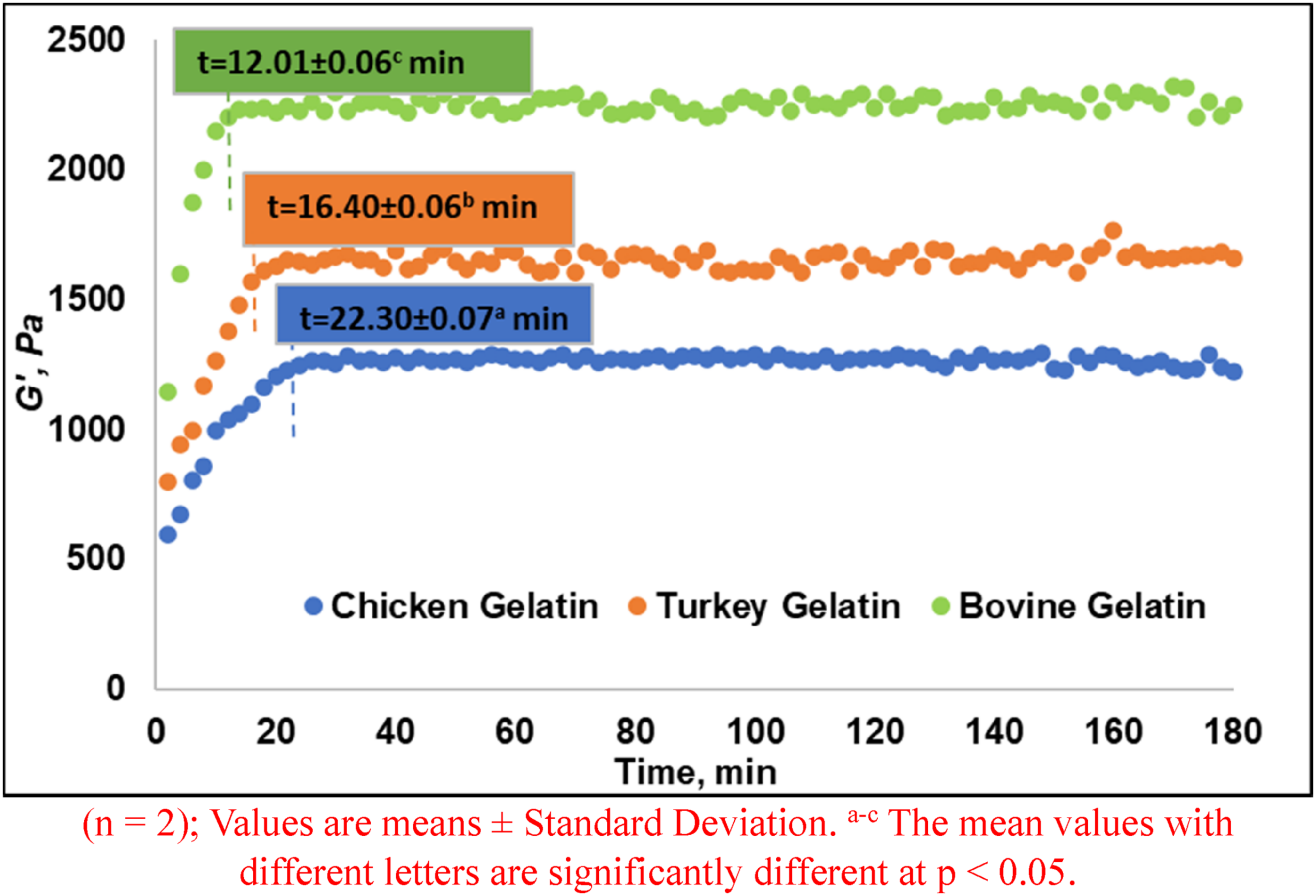
Gelation kinetics of gelatin solutions. (*n* = 2); Values are means ± standard deviation. ^a–c^The mean values with different letters are significantly different at *p* < .05.

Figure 3 illustrates the variation of *G′* at 4°C for the 3 h time sweep tests, providing essential insight into gel network formation with macro‐ or microstructural rearrangement rate over time. The kinetics of elastic character development in gelatin gels represents the transition from the primary crystallization process of gelation to the secondary crystallization process (Kuan et al., 2016). The period during which the rate of *G′* increases rapidly indicates the period during which the gel network is formed, followed by the relatively stable change with a decrease in the rate of increase of *G*′ over time (Kuan et al., 2016). It was observed that the fastest gel structure development was observed in bovine gelatin and the *G′* value of the gel structure formed was the highest. The formation of helix structure and interchain associations are more efficient in bovine gelatin that is stabilized by non‐covalent interactions. The gel network formation of turkey gelatin is faster than that of chicken gelatin and the modulus values were higher (*p* < .05). It can be concluded that the molecular interaction in the structure of the turkey gelatin produced is higher and low‐molecular‐weight components are in fewer fractions (Kuan et al., 2016). Despite these findings, it can be inferred that the more efficient factors involved in the progression of the gel network that occurred during the aging period are responsible for the highest bloom strength values of turkey gelatin. The rate of cooling, temperature, and time of maturation will affect the total amount of helix formation, and longer maturation times (aging) will result in a greater number of helixes and stabilizing junction zones (Huang et al., 2018).

### 3.8 Amino acid compositions of gelatins

Table 4 presents the amino acid composition of the gelatin samples. In accordance with other findings in the literature, chicken and turkey gelatins contain the highest amounts of glycine. This is due to the protective effect of glycine on the composition of the parent collagen protein (Venupriya et al., 2023). Therefore, the similarities in glycine content indicate that the hydrolysis of chains during pH 1 treatments showed comparable trends for chicken and turkey gelatins. The hydrolysis of the α chain when treating fish scales with an alkaline condition was performed by Venupriya et al. (2023), who reported lower glycine compared to acid‐treated gelatin. Histidine, isoleucine, methionine, ornithine, and tyrosine were the amino acids with the lowest content in gelatins, while cystine and taurine amino acids are at undetectable levels. The total proline and hydroxyproline content of gelatins is in the range of 20–23 g/100 g, which contributes significantly to collagen rigidity. It was found to be higher in turkey gelatin than in chicken gelatin, which explains the increased cross‐linking, gelation rate, and gel strength as well as the viscoelastic properties in turkey gelatin compared to chicken gelatin. Proline is an amino acid that contributes to the stability of the triple helix collagen conformation. According to a report, a repeated sequence known as Gly‐X‐Y is necessary for the efficient formation and stabilization of the collagen's triple helix structure. X and Y may correspond to any amino acid, with the average triplet comprising at least one Pro or Hyp (Qi et al., 2022). The nucleation sites of gelatin are formed by the hydrogen bonds between water molecules and the free hydroxyl groups of amino acids, which affect the strength of the resulting gel. In addition, turkey gelatin contains a higher amount of serine with a free hydroxyl group than turkey. This contributes to the formation of hydrogen bonds as mentioned above (Hafidz et al., 2011).

**TABLE 4:** Amino acid composition of chicken and turkey gelatins.

	Chicken gelatin	Turkey gelatin
Alanine	11.21 ± 0.02	11.39 ± 0.03
Arginine	10.05 ± 0.06	9.70 ± 0.01
Aspartic acid	4.80 ± 0.00	5.72 ± 0.00
Cystine	nd	nd
Glutamic acid	12.53 ± 0.00	12.34 ± 0.00
Glycine	22.41 ± 0.05	21.98 ± 0.02
Histidine	0.87 ± 0.00	0.85 ± 0.00
Isoleucine	1.24 ± 0.00	1.14 ± 0.00
Leucine	3.10 ± 0.01	3.78 ± 0.00
Lysine	4.37 ± 0.00	4.60 ± 0.00
Methionine	0.99 ± 0.00	1.05 ± 0.00
Ornithine	0.14 ± 0.00	0.20 ± 0.00
Phenylalanine	2.47 ± 0.05	2.45 ± 0.00
Proline	14.64 ± 0.00	15.21 ± 0.00
Serine	2.64 ± 0.00	3.03 ± 0.00
Threonine	2.48 ± 0.00	2.54 ± 0.00
Tyrosine	0.73 ± 0.00	0.73 ± 0.00
Valine	2.10 ± 0.00	1.87 ± 0.00
Taurine	nd	nd
Hydroxyproline	6.09 ± 0.05	7.61 ± 0.07

*Note*: (*n* = 2); Values are means ± standard deviation.

### 3.9 Peltier system microscope images

**FIGURE 4:**
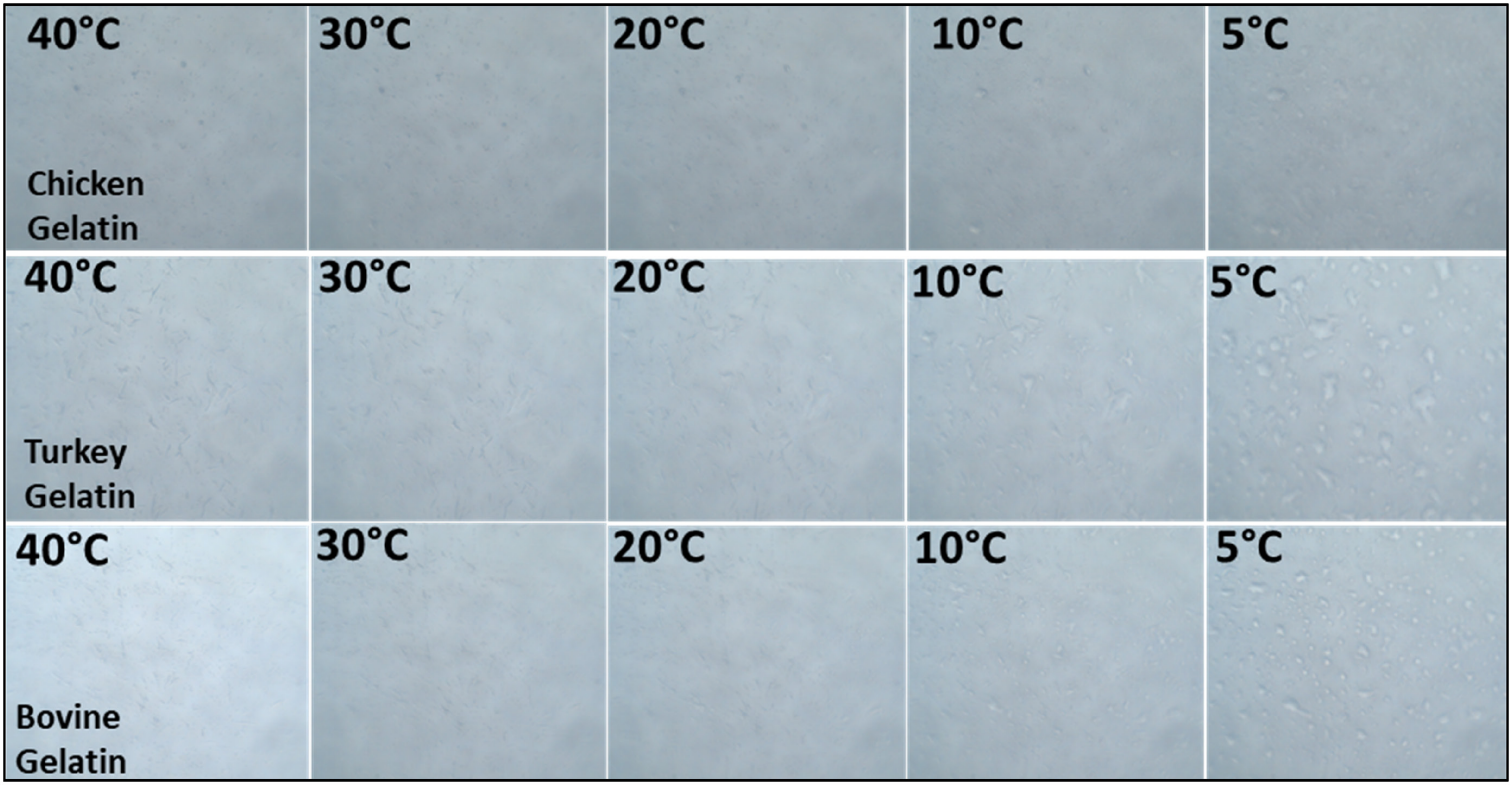
Peltier system microscope images of the gelatins.

Gelation images of gelatin samples during the period of temperature decrease were observed using peltier light microscopy (Figure 4). In line with the temperature scanning test during rheological measurements, it was observed that there was no noticeable change in the visual properties in the steps up to about 20°C, but after this temperature, the transition to a continuous gel structure occurred and, especially below 10°C, water molecules bound in the gel structure were formed. In view of these properties, the gelation profile was similar in turkey and chicken gelatins and was found to be compatible with that of bovine gelatin. This situation indicates that the applied preprocessing methods do not create any deficiency in meeting the quality criteria of the commercial gelatins (Ozcan et al., 2023). Increasing the temperature above the optimum points may result in a coarser appearance of gel images. Prolonged exposure to elevated temperatures during the extraction process can lead to the denaturation of proteins, causing the development of gel with irregular characteristics and diminished strength over time.

### 3.10 ATR‐FTIR

**FIGURE 5:**
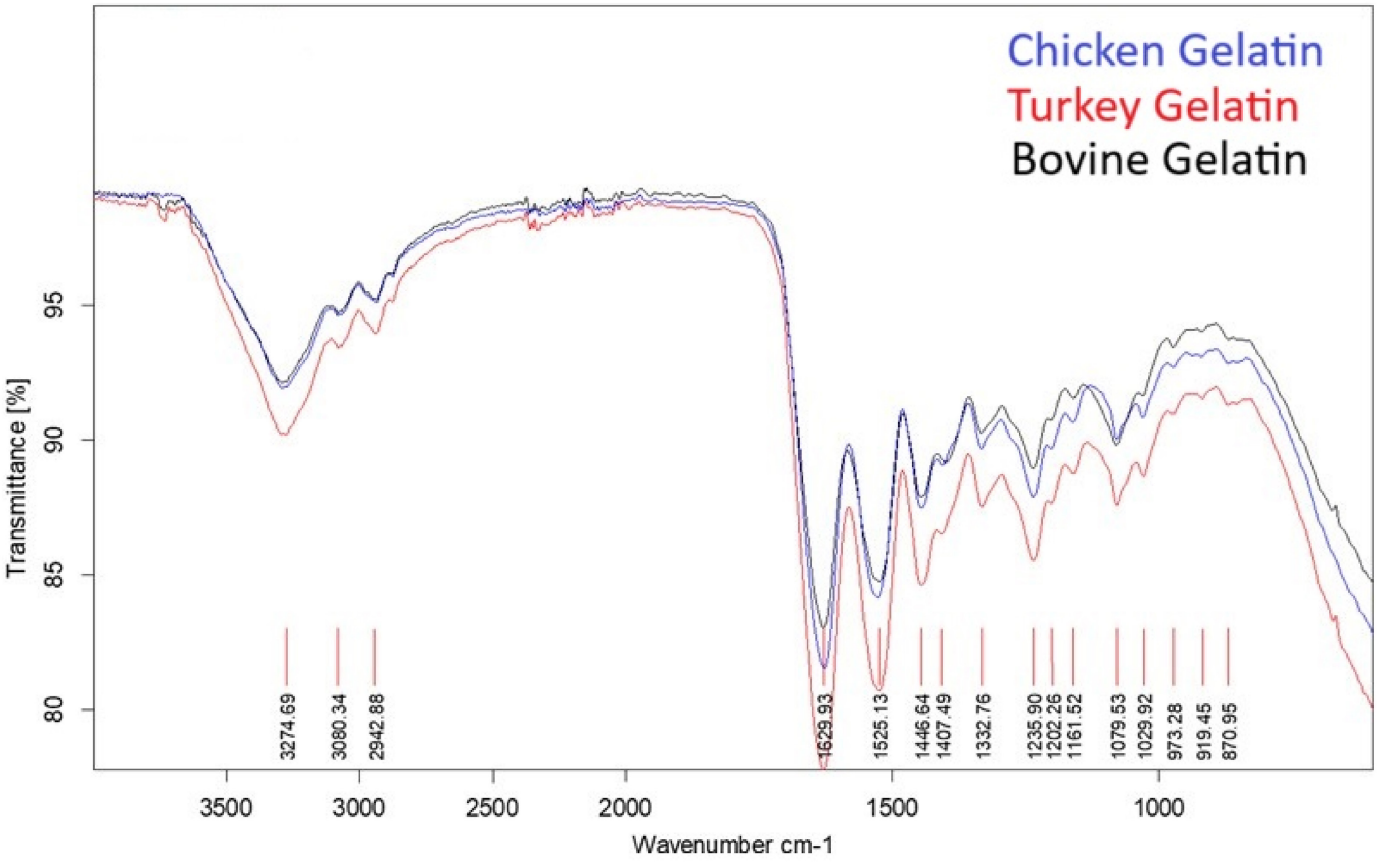
Fourier transform infrared **(**FTIR) spectra of chicken, turkey, and bovine gelatins.

Fourier transform infrared (FTIR) spectra of gelatin samples are presented in Figure 5. The FTIR analysis is important in correlating the functional groups and secondary structures with the functional behavior of gelatins (Casanova et al., 2020). The peak at approximately 1629.93 cm^−1^ is related to the asymmetric. In gelatins produced from bovine gelatin and fish waste, the amide I bands were found at 1629.39 and 1633.54 cm^−1^ wavelength, respectively, which is consistent with that given in this study (Mirzapour‐Kouhdasht et al., 2020). The sources of vibration in this band consist of 80% C=O, 10% C–N, and 10% N–H bonds (Cebi et al., 2016). A small amount of wavelength variation in the amide I band between gelatin samples indicates a difference in polypeptide conformation, but no difference in this respect was detected in the gelatins produced and bovine gelatins. At 1513 cm^−1^ wavelengths, amide II bands are observed. The chemical vibrations responsible for the formation of this band are 40% C–N vibrations and 60% N–H vibrations in the peptide groups (Ahmad et al., 2010). The lower intensity of this band is explained by the increased participation of N–H vibrations in binding to adjacent α‐chains. Specifically, the order is turkey, chicken, and bovine gelatin, wherein chicken gelatin exhibited the highest binding with α‐chains over turkey gelatin. The peak at 1235 cm^−1^ is identified as the amide III band. Amide IIІ reflects C–N vibrations and N–H vibration peaks in the amide bond. It also displays oscillatory vibrations originating from CH_2_ groups in the glycine backbone and proline side chains (Venupriya et al., 2023). When the peak intensities for this band are compared, it is seen that turkey has the highest peak intensity and bovine has the lowest peak intensity. The increased peak intensity is explained by the increased disorder in the molecular structure generated by the random helical transition of α‐helicals during extraction and the denaturation of collagen into gelatin, which results in the loss of the triple helix state (Ozcan et al., 2023). Therefore, this transformation was greater in turkey gelatin than in chicken gelatin. Another prominent peak in this region at 1079.53 is due to C–O stretching vibrations in short peptide chains (Kudo & Nakashima, 2020). Further degradation of the peptide chains is used to explain the appearance of these peaks (Ahmad et al., 2010). The distinct peak at a wavelength of 3274.69 cm^−1^ was assigned to Fermi resonances of N–H vibrations. This peak, identified as amide A, was detected at 3292.69 cm^−1^ in commercial bovine gelatins produced from fish waste (Mirzapour‐Kouhdasht et al., 2020). The shift of this band, which is generally seen at 3440–3400 cm^−1^, to approximately 3300 cm^−1^ is due to the incorporation of the N–H group into a hydrogen bond. Alternative chemical interactions, such as the N–H group of shorter peptide fragments in chicken and bovine gelatins being involved in hydrogen bonding and the formation of new covalent bonds, were found to be associated with the lower intensity of these peaks (Kemp, 2017). The peak observed at 3080.34 cm^−1^ is identified as the amide B band and is due to asymmetric C=H and NH^+3^ vibrations (Tu et al., 2015). The displacement of the amide B peak to a lower wavelength is linked to an increased interaction of the –NH_3_ group between the peptide chains, but no such difference was noticed in the gelatins in the study. As a consequence, gelatins with comparable properties to bovine gelatins, containing functional groups, were generated and the development of secondary low‐molecular‐weight degradation products was constrained during pretreatment.

### 3.11 Differential scanning calorimetry

**FIGURE 6:**
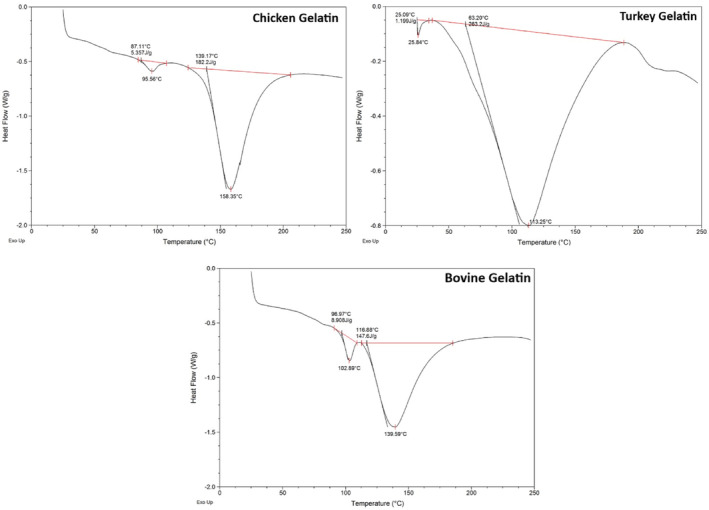
Differential scanning calorimetry (DSC) thermograms of chicken, turkey, and bovine gelatins.

Differential scanning calorimetry (DSC) thermograms of gelatin samples are given in Figure 6. Gelatin denaturation is indicated by the appearance of an endothermic peak, representing the breaking of interchain interactions leading to the fusion of peptide chains (Sila et al., 2017). The resistance to denaturation determined by the enthalpy changes and chain–helix transition temperature is referred to as the thermal stability of gelatin. Turkey gelatin had the lowest thermal transition point of 25.84°C, followed by chicken gelatin at 95.6°C and bovine gelatin at 102.89°C, indicating that thermal stability of produced gelatins is lower, whereas chicken gelatins are nearer to commercial gelatins. In previous DSC analyses of gelatins from chicken waste, this point was determined in the ranges of 58.47–63.17°C and 30.99–39.83°C (Kim et al., 2012; Mhd Sarbon et al., 2013). The lower thermal stability of turkey gelatin, despite its higher imino acid content and gel strength, is therefore attributed to the lower‐molecular‐weight compounds. This relationship is confirmed by the high peak intensity of some chemical bonds in the FTIR spectrum. Comparing the enthalpies of the second endothermic peaks, it is evident that more energy is needed for the thermal transition of turkey gelatin, which is explained by a more stable collagen conformation caused by hydrogen bonding and fewer helix–coil transitions (Venupriya et al., 2023). The molar mass of chemical species of gelatins synthesized by different ways with varying water content may be connected to the different denaturation temperatures and enthalpies found in the literature (Giacomelli da Silva et al., 2022).

## 4 CONCLUSION

This study has demonstrated that turkey and chicken gelatins can be produced with a yield of 9%–14% by denaturing MDR collagen at a maximum swelling pH of 1 followed by extraction at 80°C–6 h with a gel strength and viscosity similar to that of commercial gelatin. Due to the higher proline and hydroxyproline content, turkey gelatin was found to have a higher bloom strength than chicken gelatin and bovine gelatin had the lowest. The gelation and melting temperatures of the chicken gelatin were higher, while the time‐dependent gel network formation of the turkey gelatin was faster. The chicken and turkey gelatins had lower thermal transition temperatures than bovine gelatin, but more energy was required for their degradation due to the more stable collagen structure. Based on these results, chicken and turkey gelatins could soon be used to replace bovine and porcine gelatins in food and pharmaceutical products, potentially changing the way MDR is managed. Further studies should investigate the functional behavior of these products in jelly and marshmallow systems.

## AUTHOR CONTRIBUTIONS

**Abdullah Kurt:** Conceptualization (equal); data curation (equal); project administration (equal); supervision (equal); writing – original draft (equal); writing – review and editing (equal). **Omer Said Toker:** Conceptualization (equal); supervision (equal); writing – original draft (equal); writing – review and editing (equal). **Mehmet Akbulut:** Formal analysis (equal); investigation (equal); methodology (equal). **Hacer Coklar:** Formal analysis (equal); investigation (equal); methodology (equal). **Duygu Ozmen:** Data curation (equal); formal analysis (equal). **Yilmaz Ozcan:** Data curation (equal); formal analysis (equal). **Said Reza Hosseini:** Data curation (equal); formal analysis (equal). **Furkan Turker Sarıcaoglu:** Formal analysis (equal); investigation (equal); methodology (equal). **Guntac Demir:** Formal analysis (equal); investigation (equal); methodology (equal). **Mustafa Samil Argun:** Data curation (equal); formal analysis (equal).

## ACKNOWLEDGEMENTS

This work was supported by the Ministry of Food, Agriculture and Livestock of the Republic of Turkey (TAGEM‐20/AR‐GE/01) and the Scientific Research Projects Coordination Department of Selcuk University (project number: 22401065). The authors would like to thank TAGEM and Selcuk University for their financial support. The authors express thanks to TUBITAK (The Scientific and Technological Research Council of Turkey) for providing open‐access publication fee.

## CONFLICT OF INTEREST STATEMENT

The authors declare that they have no known competing financial interests or personal relationships that could have appeared to influence the work reported in this paper.

## ETHICS STATEMENT

Not applicable, because this paper does not contain any studies with human or animal subjects.

## Data Availability

The data that support the findings of this study are available from the corresponding author upon reasonable request.
